# Human and bacterial TatD enzymes exhibit apurinic/apyrimidinic (AP) endonuclease activity

**DOI:** 10.1093/nar/gkad133

**Published:** 2023-03-07

**Authors:** Jonathan Dorival, Brandt F Eichman

**Affiliations:** Department of Biological Sciences, Vanderbilt University, Nashville, TN, USA; Department of Biological Sciences, Vanderbilt University, Nashville, TN, USA; Department of Biochemistry, Vanderbilt University School of Medicine, Nashville, TN, USA

## Abstract

TatD enzymes are evolutionarily conserved deoxyribonucleases associated with DNA repair, apoptosis, development, and parasite virulence. Three TatD paralogs exist in humans, but their nuclease functions are unknown. Here, we describe the nuclease activities of two of the three human TatD paralogs, TATDN1 and TATDN3, which represent two phylogenetically distinct clades based on unique active site motifs. We found that in addition to 3′-5′ exonuclease activity associated with other TatD proteins, both TATDN1 and TATDN3 exhibited apurinic/apyrimidinic (AP) endonuclease activity. The AP endonuclease activity was observed only in double-stranded DNA, whereas exonuclease activity was operative primarily in single-stranded DNA. Both nuclease activities were observed in the presence of Mg^2+^ or Mn^2+^, and we identified several divalent metal cofactors that inhibited exonuclease and supported AP endonuclease activity. Biochemical analysis and a crystal structure of TATDN1 bound to 2′-deoxyadenosine 5′-monophosphate in the active site are consistent with two-metal ion catalysis, and we identify several residues that differentiate nuclease activities in the two proteins. In addition, we show that the three *Escherichia coli* TatD paralogs are also AP endonucleases, indicating that this activity is conserved across evolution. Together, these results indicate that TatD enzymes constitute a family of ancient AP endonucleases.

## INTRODUCTION

DNA is under constant attack from endogenous and exogenous sources. Abasic sites, also known as apurinic/apyrimidinic (AP) sites, are the most frequent type of DNA damage (1–3). AP sites result from spontaneous depurination and depyrimidination, ionizing radiation and oxidative damage, and enzymatic removal of damaged or mismatched nucleobases by DNA glycosylases as the first step of the base excision repair (BER) pathway (4–8). AP sites are mutagenic and pose a threat to genome stability by their ability to block or otherwise miscode DNA synthesis by polymerases (9–11). Moreover, the ring-opened, aldehyde form of an AP site can react with both DNA and proteins to generate interstrand DNA and DNA–protein crosslinks, and is susceptible to strand breaks through base-catalyzed β-elimination (1,12–14).

Several pathways exist to repair AP sites. In double-stranded (ds) DNA, AP sites are repaired by BER, wherein an AP endonuclease cleaves the phosphodiester bond 5′ to the AP site to generate a free 3′-hydroxyl used for gap-filling synthesis by a DNA polymerase (5). *Escherichia coli* possess two AP endonucleases, Exonuclease III (*xth* or ExoIII) and Endonuclease IV (*nfo* or EndoIV), which carry out 90% and 10% of the cellular AP endonuclease (AP endo) activity, respectively (15–17). ExoIII, and to a lesser extent EndoIV, also contains 3′-5′ exonuclease (3′-exo) activity (17,18). Similarly, humans contain two AP endonucleases, APE1 and APE2. APE1 functions as part of the BER pathway and accounts for the majority of AP endo activity in humans (6,19–21), whereas APE2 displays comparatively weak AP endo activity (22). APE1 and APE2 also contain a range of 3′-5′ exonuclease and 3′-phosphodiesterase activities important for repair of oxidative DNA damage and blocked 3′-ends (23,24). In the context of single-stranded (ss) DNA, AP sites are not repaired by strand scission but instead are covalently modified by SRAP proteins or non-covalently bound by the Shu complex in yeast (25), likely as a way to protect cells from a double-strand break until the AP site can be repaired by a yet unknown mechanism (25–29).

TatD proteins are a large family of 3′-5′ exonucleases conserved from bacteria to humans that have been implicated in DNA repair and other important DNA processing functions. TatD was first identified within the *E. coli tat* operon that allows the transport of fully folded proteins from the cytoplasm to the periplasm (30–32). However, TatD and its two paralogs, YjjV and YcfH, were dispensable for Tat-dependent protein transport in *E. coli* (33). Consistent with its similarity to the polymerase and histidinol phosphatase superfamily of metal-dependent phosphoesterases (34), *E. coli* (Ec) TatD (also named ExoXI) was found to exhibit magnesium-dependent deoxyribonuclease activity (33) and was later characterized as a 3′–5′ exonuclease with activity on ssDNA and RNA substrates (35). EcTatD has been implicated in oxidative DNA damage repair as *tatD* deletion sensitized *E. coli* to hydrogen peroxide (35). TatD from *Saccharomyces cerevisiae* (Sc) also displays 3′-exo activity, as well as a non-specific endonuclease activity (36). Unlike the *E. coli* enzyme, ScTatD showed greater nuclease activity on dsDNA compared to ssDNA, and deletion of ScTatD *increased* cell survival after H_2_O_2_ treatment, suggesting a role in apoptosis (36). Similarly, TatD homologs from *Caenorhabditis elegans*, *Trypanosoma brucei*, and *Leishmania donovani* have also been implicated in degradation of apoptotic DNA (37,38). The zebrafish TATDN1 ortholog was shown to cleave supercoiled DNA and to decatenate DNA circles into linear and nicked open circular DNA (39). Knockdown of TATDN1 in zebrafish embryos delayed cell cycle progression and led to formation of polyploidy, aberrant chromatin structures, and abnormal eye development (39). More recently, TatD orthologs were found to operate as virulence factors in *Plasmodium* and trypanosome parasites by hydrolyzing host neutrophil extracellular traps induced by the parasites (40,41).

Humans contain three human TatD paralogs, TATDN1, TATDN2 and TATDN3. Although several reports have focused on possible roles for their RNA transcripts (42–47), to our knowledge the proteins have not been characterized. Here, we report that TATDN1 and TATDN3 contain AP endo activity in addition to their 3′-exo activity. They show preferential AP endo activity on dsDNA substrates and 3′-exo activity on ssDNA. The 3′-exo activity is sensitive to the metal ion present in the active site, whereas the AP endo activity is observed in a variety of divalent metal cofactors. A crystal structure of TATDN1 bound to 2′-deoxyadenosine 5′-monophosphate (dAMP) revealed two metal ions bound in the active site, which we corroborated biochemically, providing insight into the mechanism of catalysis by TatD enzymes. We also show that the AP endo activity of TatD enzymes is conserved across evolution as the three *E. coli* paralogs TatD, YjjV and YcfH also exhibit this activity.

## MATERIALS AND METHODS

### Sequence alignment and phylogenetic analysis

Sequences of the human TATDN1, TATDN2 and TATDN3 were used for BLASTp (48) searches to find eukaryotic homologs, and TatD, YjjV and YcfH were used to find the prokariotic homologs. All sequences were verified manually to select only the full-length isoforms. Sequence alignment was performed using Clustal Omega (49) and illustrated with ESPript (50). For the phylogenetic tree, MEGA (51) was used to perform the sequence alignment using the MUSCLE algorithm. Maximum likelihood analysis was performed with IQtree using default parameters (52) and the phylogenetic tree was generated using Interactive Tree of Life (53), using the human APE1 sequence to root the tree.

### Protein purification

The genes encoding human TATDN1 and TATDN3 and *E. coli* TatD, YjjV and YcfH were synthesized by GenScript. All except TatD were ligated into a modified pET-27 expression vector encoding an N-terminal Rhinovirus 3C-cleavable hexahistidine-SUMO fusion tag. Plasmids were transformed into *E. coli* BL21 (DE3) cells and grown in Luria Bertani (LB) medium supplemented with 40 mM sodium phosphate pH 7.7. When the cultures reached an *A*_600_ of 0.8, protein expression was induced by addition of IPTG (final concentration of 0.1 mM) followed by incubation at 15°C for 12–18 h. Cells were harvested by centrifugation and the resulting cell pellets resuspended in lysis buffer (50 mM sodium phosphate pH 7.7, 500 mM NaCl, 10% mannitol) and lysed by sonication. Cell debris was removed by centrifugation at 45 000 × g for 30 m. Nucleic acids were precipitated by adjusting NaCl concentration of the supernatant to 1 M and adding polyethylenimine (PEI) to a final concentration of 0.1%. After centrifugation, ammonium sulfate was added to the supernatant at 70% saturation. Samples were centrifuged for another 30 m and the pellet was resuspended in buffer A (lysis buffer supplemented with 40 mM imidazole) and injected onto an Ni-NTA column. The column was washed with buffer A and protein eluted using buffer B (lysis buffer supplemented with 500 mM imidazole). Fractions were pooled and the hexahistidine-SUMO tag was removed by overnight cleavage at 4°C while dialyzing against the lysis buffer to remove imidazole. The sample was reinjected onto a Ni-NTA column and the flow-through collected. The protein was concentrated using an Amicon Ultracel-10 (Merck Millipore) and injected onto a Superdex 200 (16/60 or 26/60 depending on the quantity of protein) equilibrated in buffer S (30 mM HEPES pH 7.7, 300 mM NaCl, 5% glycerol). Fractions containing the protein were pooled and concentrated by diafiltration using an Amicon Ultracel-10 (Merck Millipore). Aliquots were flash frozen in liquid nitrogen and stored at −80°C.

The *E. coli* TatD gene was cloned into a modified pET-21 expression vector to produce an untagged protein. Protein was expressed as above. Cell pellets were resuspended and lysed in 30 mM HEPES pH 7.5 and 300 mM NaCl, nucleic acids precipitated with 0.1% PEI, and protein precipitated with ammonium sulfate at 65% saturation. The pellet was resuspended in buffer containing 20 mM HEPES pH 7.7 and 1 M ammonium sulfate and injected onto a phenyl-sepharose HP column (Cytiva). The flow-through was collected and dialyzed overnight against buffer containing 20 mM HEPES pH 7.7 and 75 mM NaCl. Protein was injected onto a Q-sepharose HP column (Cytiva) and eluted with a 0–1 M NaCl gradient, followed by size exclusion on a Superdex 200 16/60 equilibrated in 20 mM HEPES pH 7.7 and 200 mM NaCl. The protein was then concentrated and frozen as above.

Mutants were generated using the Q5 site-directed mutagenesis kit (New England Biolabs) and purified the same as the wild-type proteins.

### Nuclease activity

Oligonucleotides were purchased from Integrated DNA Technologies. The sequences used are provided in Supplementary Table S1. Sequences containing natural AP sites were generated by reacting 50 μl of 50 μM dsDNA uracil-containing oligonucleotide with 5 U uracil DNA glycosylase (New England Biolabs) for 60 m at 37°C. Oligonucleotides were resuspended and annealed in 10 mM MES pH 6.5 and 40 mM NaCl and diluted into reaction buffer (20 mM *N*-(2-hydroxyethyl)piperazine-*N*′-(3-propanesulfonic acid (EPPS) pH 8.0, 50 mM NaCl and 2.5% glycerol). Nuclease reactions were carried out at 37°C and contained 10 μM protein, 100 nM DNA, and reaction buffer supplemented with either 10 mM MgCl_2_ (exonuclease reactions) or 5 mM CaCl_2_ (AP endonuclease reactions). Experiments to test activity in the presence of different cofactors were carried out under the same conditions but supplemented with either 0.5 mM ZnCl_2_, 3 mM MnCl_2_, 1 mM NiCl_2_ or 10 mM EDTA. To quench the reactions, 8 μl aliquots were mixed with 10 μl loading buffer (10 units proteinase K, 80% formamide, 10 mM EDTA pH 8.0, 2 mg/mL orange G, and 1 mg/ml xylene cyanol). Samples were heated at 70°C for 10 m and subjected to denaturing polyacrylamide gel electrophoresis (PAGE) on 20% acrylamide/8M urea sequencing gels. Fluorescence from the FAM-labeled DNA was detected using a Typhoon Trio variable mode imager (GE Healthcare). Rate constants were calculated by fitting data to a single-exponential. Experiments were performed in triplicate.

### Thermal denaturation

Protein stability was monitored by differential scanning fluorimetry using a CFX96 real time-PCR instrument (BioRad) operating in FRET mode. Samples (25 μl) contained 15 μM protein, 30 mM HEPES pH 7.5, 100 mM NaCl, 0.5× SYPRO orange stain (Invitrogen), and 0–10 mM ZnSO_4_, MgCl_2_, or CaCl_2_. Fluorescence was measured from 25-μl solutions in 0.2 ml thin-wall 96-well PCR plates (BioRad) sealed with transparent Microseal B adhesive sealer (BioRad). Measurements were taken at increasing temperature from 20–80°C in steps of 0.5°C with an equilibration time of 10 s at each step. Experiments were performed in triplicate.

### Crystal structure determination

TATDN1 at 1.2 mM (40 mg/ml) was incubated with 50 mM dAMP (Sigma-Aldrich, pH adjusted to 8.0 with NaOH) and 2 mM ZnSO_4_ on ice for 15 m. Crystals were grown by sitting drop vapor diffusion by mixing 1 μl protein with 1 μl crystallization buffer (19% PEG 8000, 100 mM HEPES pH 7.0). Crystals were soaked briefly in crystallization buffer containing 25% glycerol before being flash frozen in liquid nitrogen. X-ray diffraction data were collected at beamline ID-D (λ = 1.12705 Å) at the Advanced Photon Source (Argonne National Laboratory) and processed using XDS (54). Phases were determined by molecular replacement with MoRDa (55) using the structure of apo TATDN1 (PDB ID 2XIO) as the search model. Model building and refinement were performed with Coot (56) and Refmac (57). Feature enhanced maps (58) were generated to position the ligands with minimal model bias. Anomalous difference Fourier maps for Zn^2+^ were generated in PHENIX (59) from the same X-ray data reprocessed to treat Friedel pairs as separate reflections, since Zn^2+^ has a strong theoretical absorption signal at 1.12705 Å (peak absorption is at 1.2837 Å). Structures were validated using MolProbity (60). Data collection, refinement, and validation statistics are presented in Supplementary Table S2. Structure factors and coordinates have been deposited in the Protein DataBank under accession number 8EFG. All protein structure figures were prepared using the program PyMol (Schrödinger, LLC).

### TATDN1 DNA binding model

DNA docking experiments were performed using HADDOCK 2.4 (61). Input files consisted of the structure of TATDN1 and AP-DNA from the EndoIV/AP-DNA crystal structure (PDB ID 2NQJ) (62). Since the AP site was not recognized by HADDOCK, it was converted to deoxycytosine (dC) prior to docking. Heteroatoms, with the exception of the two zinc ions, were also removed. Unambiguous distance restraints were generated to anchor the zinc atoms in the active site. DNA was docked as a rigid body using residues Arg151, Trp225 and the two zinc ions as protein-DNA interaction constraints. The most energetically favorable model had the dC flipped out of the DNA helix and pointed toward the active site, which precluded interaction between the DNA backbone and the zinc ions. The cytosine base was removed from this docked model and the resulting AP-DNA further manually positioned so that the phosphodiester bond 5′ to the AP site was within 5 Å of the zinc ion without introducing steric clashes. A cycle of energy minimization was then performed using the YASARA server (63).

## RESULTS

### TatD proteins are found in three phylogenetically distinct clades

**Figure 1.**
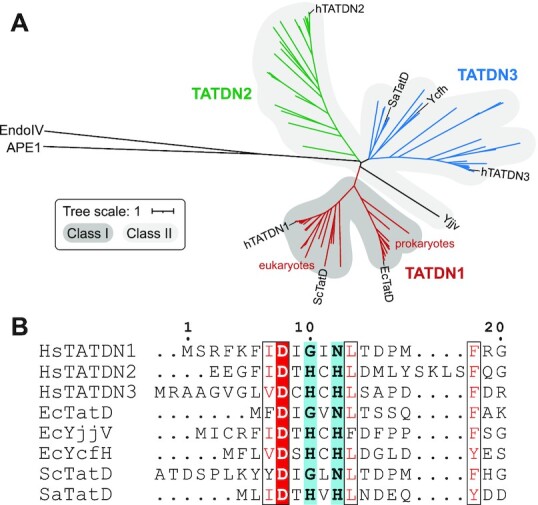
Phylogenetic study of TatD proteins. (**A**) Phylogenetic analysis of eukaryotic and prokaryotic TatD proteins. The tree was rooted using the sequence of human APE1. EndoIV, which contains a similar protein fold as TatD, is shown for comparison. Branch length represents the number of substitutions per site (the length corresponding to 1 substitution/site is shown in the inset). Clades representing TATDN1, TATDN2 and TATDN3 are colored red, green, and blue. Class I and II proteins are shaded dark and light grey, respectively. (**B**) Sequence alignment of the N-terminal region of TatD orthologs from human, *E.coli* (Ec), *Saccharomyces cerevisiae* (Sc), and *Staphylococcus aureus* (Sa). The (G/A)xN (Class I) and HxH (Class II) active site motifs are highlighted blue.

To determine the evolutionary relationship of the human TatD proteins, we performed a phylogenetic analysis of TATDN1, TATDN2 and TATDN3. The proteins clustered into three distinct clades, each of which contained proteins well represented in eukaryotes and prokaryotes (Figure 1A, Supplementary Figure S1A). TATDN1 proteins are ubiquitous; we did not find any phyla that lacked a TATDN1 ortholog. TATDN2 and TATDN3 were found in most organisms except many protozoans, which instead possess several copies of TATDN1, and fungi outside of the phyla Piromyces, Zygomycota, Glomeromycota and Mucoromycota. Vertebrates possess all three enzymes with the exception of birds, which lack TATDN2. The TATDN1 clade showed the most sequence conservation, as represented by the tree branch lengths, with clear sub-branches for eukaryotic and prokaryotic orthologs. The TATDN2 clade was the most heterogeneous, mainly because in higher eukaryotes these proteins possess an extended N-terminal region that often contains a nuclear localization signal.

In addition to the evolutionary distinction of the three proteins, they can be categorized into one of two subfamilies—previously referred to as Groups I and II—on the basis of a unique N-terminal sequence motif that forms part of the active site (35). TATDN1 contains a (G/A)xN motif (Group I) and TATDN2 and TATDN3 contain an HxH motif (Group II) (Figure 1B, Supplementary Figure S1B). The three *E. coli* paralogs (TatD, YjjV, YcfH) partition the same way; TatD is in Group I and YjjV and YcfH are in Group II (Figure 1B).

### Human TATDN1 and TATDN3 exhibit AP-endonuclease and 3′-exonuclease activities

**Figure 2.**
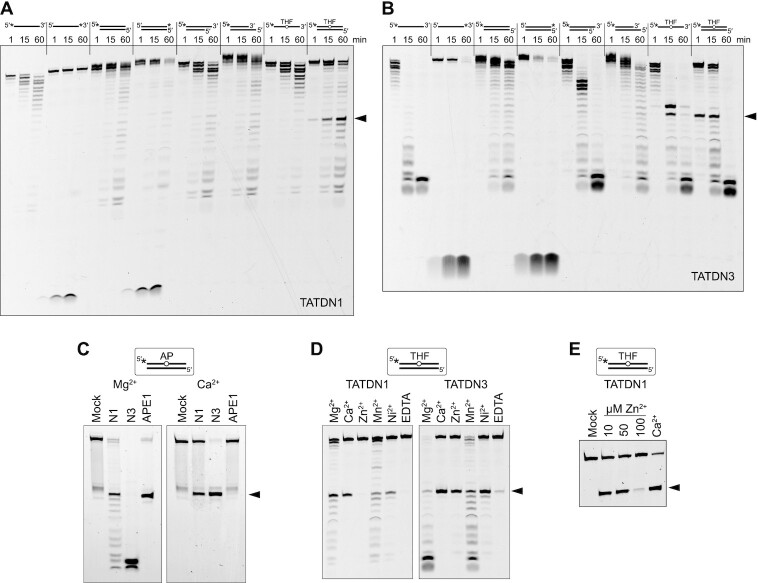
TATDN1 and TATDN3 exhibit AP endonuclease and 3′-5′ exonuclease activities. (**A**, **B**) Nuclease activities of TATDN1 (**A**) and TATDN3 (**B**) on various DNA substrates, visualized by denaturing gel electrophoresis. Reactions contained 10 mM MgCl_2_. The band resulting from AP endonuclease activity is indicated by a black triangle. THF, tetrahydrofuran. (**C**) Comparison of AP endonuclease activities of human TATDNs and APE1 on a natural AP site in the presence of either MgCl_2_ (left gel) or CaCl_2_ (right gel). The reactions were performed at 37°C for 2 h using 10 μM TATDN1/3 or 10 nM APE1. (**D**) Metal dependence of TATDN1 and TATDN3. Reactions contained 10 μM protein and either 10 mM MgCl_2_, 5 mM CaCl_2_, 0.5 mM ZnCl_2_, 3 mM MnCl_2_, 1 mM NiCl_2_, or 10 mM EDTA. (**E**) AP endo activity of TATDN1 (10 μM) at Zn^2+^ concentrations below 100 μM. Mock, no-enzyme control. Reaction in the presence of 5 mM Ca^2+^ (far right lane) is included as a positive control.

Several prokaryotic and eukaryotic TatD proteins have been characterized as deoxyribonucleases in general, and in most cases as 3′-exonucleases, with varying substrate specificities (33,35–37,39,40,64,65). We tested the nuclease activities of human TATDN1 and TATDN3 on short (25-mer) oligodeoxynucleotide substrates to assess exonuclease polarity and preference for single-stranded (ss) versus double-stranded (ds) DNA. We were unable to test TATDN2 because of protein solubility issues. DNA substrates contained a fluorescein label on either the 5′ or 3′ end and dsDNA substrates contained either 0 or 8 overhanging nucleotides of ssDNA. Using Mg^2+^ as a cofactor, both enzymes displayed 3′-exo activity on all substrates tested based on the ladder of single-nucleotide digestion products generated from 5′-labeled substrates and the single, low-molecular weight product generated from 3′-labeled substrates, which is consistent with removal of the 3′-FAM nucleotides (Figure 2A, B). Moreover, both enzymes exhibited a preference for ssDNA over dsDNA based on the faster rates of substrate disappearance and accumulation of exonuclease products from ssDNA and overhanging 3′-ends as compared to dsDNA and recessed 3′-ends (Figure 2A, B). TATDN1′s preference for ssDNA was modest (Figure 2A). In contrast, TATDN3 showed a strong preference for ssDNA, most notable in the 3′-overhang substrate, in which the ssDNA overhang was rapidly degraded until the duplex region was encountered (Figure 2B). In general, TATDN3 showed greater activity than TATDN1 on all substrates tested.

TATDN1 and TATDN3 adopt a triosephosphate isomerase (TIM) barrel fold found in many metallo-dependent hydrolases, including the *E. coli* AP endonuclease EndoIV (35,66,67) (Supplementary Table S3). We therefore tested the ability of TATDN1 and TATDN3 to incise AP-DNA. Using 5′-FAM-labeled 25-mer substrates containing a tetrahydrofuran (THF) abasic site analog at position 13, in the presence of Mg^2+^ both TATDN1 and TATDN3 generated a product band at the expected size for cleavage at the THF, most notably in the context of dsDNA (Figure 2A, B). The band corresponding to cleavage of the AP site appeared and accumulated faster than the bands arising from the exonuclease activity, suggesting that AP endonuclease is the major nuclease activity in dsDNA. In contrast, neither enzyme had significant AP endo activity on ssDNA. Cleavage at the AP site in ssDNA was only visible from TATDN3 reactions as a secondary product likely from stalling of 3′-exo activity at the THF and the nucleotide 3′ to the AP site (Figure 2B). TATDN1 did not produce a band indicative of incision of THF or stalling of 3′-exo in ssDNA (Figure 2A). The preference of AP endo activity from dsDNA over ssDNA is similar to that of APE1 (21,68) and in cells would help avoid a single-strand break. We verified that the observed AP endo and 3′-exo activities were not the result of ExoIII or EndoIV contamination from *E. coli*, since we observed the same activities from TATDN proteins purified from the *xth*^−^*nfo*^−^ strain BH110(DE3) (69) (Supplementary Figure S2A). Moreover, the preferential ssDNA 3′-exo activities and dsDNA AP endo activities are different from the exo and AP endo activities of ExoIII and EndoIV (Supplementary Figure S2B).

We next compared the AP endo activity of the human TATDN and APE1 enzymes for natural AP sites generated from DNA glycosylase excision of uracil from dsDNA (Figure 2C). TATDN1 generated a predominant AP incision product and a ladder of single-nucleotide products consistent with subsequent 3′-5′ exonuclease degradation of the remaining DNA. Under the same conditions, TATDN3 completely degraded the substrate over the course of an hour, similar to its activity for THF-DNA (Figure 2B, C). Thus, in the presence of Mg^2+^, the TATDNs display both AP endo and 3′-exo activities whereas APE1 mainly has AP endo activity (70,71). In contrast, in the presence of Ca^2+^, the TATDNs showed AP endo activity exclusively (Figure 2C). Consistent with previous studies (72,73) no Ca^2+^ dependent APE activity was observed (Figure 2C). Using Ca^2+^ as a tool to monitor AP endo activity, we found that in the context of dsDNA, TATDN3 is 5-fold more active than TATDN1, and that neither enzyme incises AP sites in ssDNA (Supplementary Figure S3), consistent with the results from Mg^2+^ reactions (Figure 2A, B).

TatD proteins have been characterized only as Mg^2+^-dependent nucleases (33,35–37,39,40,64,65), although Mg^2+^, Zn^2+^, Mn^2+^ and Ni^2+^ have been observed in the active sites of several TatD structures in the PDB (Supplementary Table S3). Based on this and the modulation of AP endo and 3′-exo activities by Ca^2+^, we characterized the effects of Zn^2+^, Mn^2+^ and Ni^2+^ on the AP endo activities of TATDNs. Using the THF-dsDNA substrate, both enzymes exhibited AP endo and 3′-exo activities in the presence of Mg^2+^ and Mn^2+^ (Figure 2D). Exonuclease activity was suppressed in the presence of Ca^2+^, Zn^2+^ and Ni^2+^, whereas all three of these cofactors supported AP endo activity. TATDN1 did not exhibit either nuclease activity at the high concentration of Zn^2+^ used (0.5 mM) in these experiments. However, we observed a low level of AP endo activity by TATDN1 at Zn^2+^ concentrations <100 μM (Figure 2E), and confirmed by differential scanning fluorimetry that inactivity was a result of destabilization of the protein at Zn^2+^:TATDN1 ratios >4 (Supplementary Figure S4). Thus, all five cofactors tested supported AP endo activity, whereas 3′-exo activity was only observed with Mg^2+^ and Mn^2+^.

The TATDN cofactor specificity is different from that of *E. coli* ExoIII and EndoIV, which exhibit both AP endo and exo activities (ExoIII) or only AP endo activity (EndoIV) in the presence of all metals tested (Supplementary Figure S2C), providing further evidence against a contaminating activity in our enzyme preparations.

### TATDN1 likely employs two-metal catalysis

Divalent cations serve as cofactors in deoxyribonucleases by activating a water nucleophile and by stabilizing the transition state that develops on the DNA phosphate (74). We investigated the roles of the metal cofactors in the human TATDN proteins, focusing on TATDN1 since the number of divalent metal ions employed by the Class I proteins is not clear despite the available crystal structures (Supplementary Table S3). Whereas Group II proteins typically coordinate two metal ions with the help of the additional two histidine residues of the HxH motif, Class I structures either lack bound metal ion or contain a single cation in various positions, leading to the proposal that Class I proteins can accommodate up to three metal ions (35). Adding to the uncertainty is that the structure of *E. coli* TatD bound to a trinucleotide did not contain any cofactors (35).

**Figure 3.**
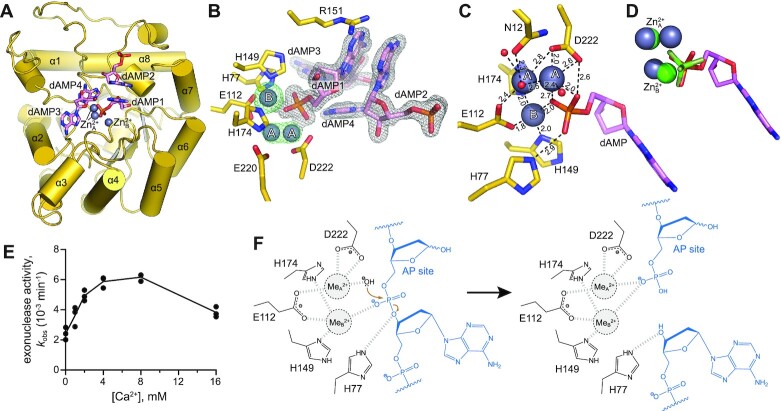
Structural characterization of TATDN1. (**A**) Crystal structure of human TATDN1 with four dAMP molecules (sticks) and two zinc ions (Zn_A_^2+^ and Zn_B_^2+^, grey spheres) bound in the active site. Zn_B_^2+^ is present at a full occupancy, while Zn_A_^2+^ occupies two positions. (**B**) 2Fo – Fc electron density map (grey mesh) is contoured at 1σ and superimposed only on the dAMP molecules (pink carbons). Zn anomalous difference map (green mesh) is contoured at 6σ and superimposed on the zinc ions (labeled A and B). (**C**) Coordination of the two Zn^2+^ ions in the active site of TATDN1. Distances are in Ångstrom. (**D**) Superimposition of ligands bound to TATDN1 and TATDN3 (PDB ID 2Y1H). The two zinc ions of TATDN1 (grey) and TATDN3 (green) occupy the same sites, and the dAMP phosphate bound to TATDN1 (pink sticks) overlays with the phosphate ion bound to TATDN3 (green sticks). (**E**) Metal complementation assay, in which TATDN1 exonuclease activity was measured at 2 mM Mg^2+^ and various concentrations of Ca^2+^. Rates are plotted from three independent experiments, with the line representing the mean. (F) Putative catalytic mechanism of TATDN1 AP endonuclease activity based on the crystal structure. Me^2+^, divalent metal cation.

To elucidate the number of catalytic metal ions in Class I proteins, we determined a crystal structure of human TATDN1 bound to dAMP in the presence of Zn^2+^ (Figure 3A). The structure was determined by molecular replacement using the unliganded structure of TATDN1 (PDB ID 2XIO) as a search model. Two zinc ions (Zn_A_^2+^ and Zn_B_^2+^) and four dAMP molecules were clearly visible in the electron density (Figure 3B). We verified the identity of the Zn^2+^ ions using anomalous difference Fourier analysis (Figure 3B). Two well-defined nucleotides (dAMP1 and dAMP2) are stacked against each other and the other two (dAMP3 and dAMP4) are only partially defined at lie the edge of the active site. Importantly, dAMP1 is oriented with its phosphate pointing into the active site and coordinated by both Zn^2+^ ions. Zn_A_^2+^ partially occupies two alternative positions and is coordinated by Glu112, Asp222, the dAMP phosphate, and several water molecules (Figure 3C). Zn_B_^2+^ was refined to full occupancy and is coordinated by residues Glu112, His149, and His174, as well as by an oxygen of the dAMP1 phosphate (Figure 3C). The presence of dAMP and Zn^2+^ did not significantly affect the protein structure; the r.m.s.d. between our structure and apo TATDN1 (PDB ID 2XIO) was 0.323 Å (Supplementary Figure S5). The two metals and the dAMP phosphate occupy the same positions as two Zn^2+^ ions and a phosphate ion present in a structure of TATDN3 (PDB ID 2Y1H) (Figure 3D, Supplementary Figure S5). Comparison of our TATDN1/dAMP/Zn^2+^ structure to the that of EcTatD bound to a trinucleotide with no cofactor (PDB ID 4PE8, (35)) shows that the active site residues are in the same location, with the exception of Asn12 (Asn7 in EcTatD), which coordinates Zn_A_^2+^ in our structure (Supplementary Figure S5). Thus, both metal ions are necessary to fully engage nucleic acid in the active site. These data also indicate that the (G/A)xN motif of Class I proteins supports binding of two metal ions with nucleic acid present in a catalytic orientation.

To determine whether two metals are required for catalysis, we monitored the exonuclease activity of TATDN1 at increasing Ca^2+^ concentrations and a fixed Mg^2+^ concentration (2 mM), which does not support maximal activity. On its own, calcium is unable to support the exonuclease activity of these enzymes. Therefore, if only one metal ion were bound in the active site, calcium would reduce exonuclease activity by competing with and displacing magnesium. However, if two or more metals were required for catalysis, addition of calcium to the suboptimal magnesium concentration would generate a mixed-ion active site and potentially promote exonuclease activity up to the concentration at which the magnesium is displaced (75–77). Consistent with a two-metal ion mechanism, we observed a bell-shaped titration curve whereby addition of calcium promoted the exonuclease activity of TATDN1 up to about 8 mM, after which higher concentrations inhibited activity (Figure 3E). This result is consistent with the hypothesis that TATDN1 binds at least two metal ions in its active site during catalysis, consistent with our crystal structure. Based on these data, we propose a catalytic mechanism for AP endo activity in which one metal ion (equivalent to Zn_A_^2+^ in our structure) activates a water molecule to perform a nucleophilic attack on the phosphate group of the DNA, and the second metal ion (Me_B_^2+^) stabilizes the negative charge on the phosphate as well as the developing charge in the pentacovalent phosphate transition state (Figure 3F). While we did not observe a potential water nucleophile in our crystal structure, replacing the more solvent exposed of the two Zn_A_^2+^ positions with a water molecule would position it for catalysis without disturbing the two-metal ion geometry.

### Cofactor and DNA binding residues important for nuclease activities

**Figure 4.**
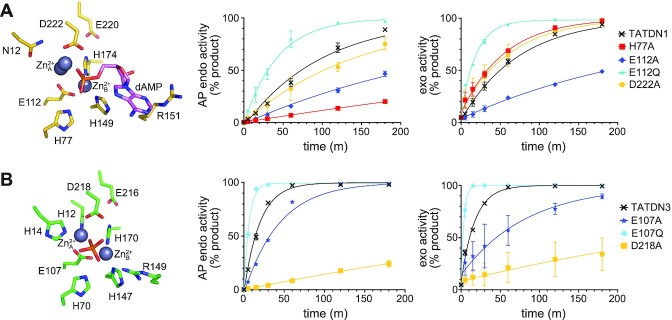
Mutational analysis of TATDN1 and TATDN3. (**A**, **B**) AP endonuclease (left plots) and exonuclease (right plots) activity of TATDN1 (**A**) and TATDN3 (**B**) mutants. Crystal structures of TATDN1/dAMP and TATDN3/PO_4_^3−^ (PDB ID 2Y1H) are shown to the left. AP endonuclease activity was measured on a 25-mer THF-containing DNA duplex in the presence of 5 mM CaCl_2_ and the exonuclease activity was measured using an unmodified 25-mer DNA duplex in the presence of 10 mM MgCl_2_. Data are represented as the mean ± SD (*n* = 3). Representative gels from which these data were quantified are shown in Supplementary Figure S7.

To gain further insight into TATDN1 catalysis, we performed a mutational analysis of residues involved in metal coordination on both AP endonuclease and exonuclease activities. AP endo activity was measured using THF-dsDNA in the presence of Ca^2+^ and 3′-exo activity was measured using unmodified dsDNA in the presence of Mg^2+^. Of the residues tested, only alanine substitution of Glu112 significantly reduced both activities (Figure 4A, Supplementary Table S4). The E112A mutation likely disrupts cofactor binding as Glu112 interacts with both Zn_A_^2+^ and Zn_B_^2+^ (Figure 3C). Interestingly, an E112Q mutation significantly increased both AP endo and 3′-exo activities. We speculate that the glutamine retains binding of the metal ion while promoting affinity for DNA by removing a negative charge present in the wild-type enzyme. Alanine and glutamine substitutions of the analogous residue in TATDN3 (Glu107) had the same inhibitory and stimulatory effects as in TATDN1 (Figure 4B, Supplementary Table S5), indicating that this residue plays the same role in Class I and II enzymes. Alanine substitution of Asp222, the only other cofactor-binding carboxylate side chain in the TATDN1 active site, did not significantly affect either AP endo or 3′-exo activity (Figure 4A, Supplementary Table S4), likely because it only interacts with Zn_A_^2+^. In contrast, the equivalent D218A mutation in TATDN3 reduced both activities by 30-fold (Figure 4B, Supplementary Table S5). Unlike TATDN1 Asp222, TATDN3 Asp218 makes a unique interaction with His12 of the HxH motif, which is not present in TATDN1. Finally, alanine substitution of TATDN1 Glu220, which sits just outside of the metal coordination sphere, did not affect either activity (Supplementary Figure S6).

We also tested alanine mutants of the histidine residues in the TATDN1 active site. Of these, only H77A had a significant effect. Strikingly, the H77A mutant reduced AP endonuclease activity 10-fold without affecting the exonuclease activity (Figure 4A, Supplementary Table S4). This result is consistent with the fact that His77 does not coordinate a metal cofactor but forms a hydrogen bond to the phosphate oxygen, and thus likely positions the substrate for catalysis. Alternatively, this residue may be sensitive to the nature of the metal ion in such a way that the alanine substitution reduces the affinity for Ca^2+^ greater than Mg^2+^. In contrast, mutation of either of the other two histidines in the active site (H149A and H174A), each of which form only a monovalent interaction with Zn_B_^2+^, only reduced the AP endonuclease activity 2-fold and did not affect the exonuclease activity (Supplementary Figure S6, Supplementary Table S4). Taken together, these data show that the two nuclease activities are catalyzed by specific cofactor or DNA binding residues, and that the differences between TATDN1 and TATDN3 activities are influenced by the (G/A)xN and HxH motifs.

**Figure 5.**
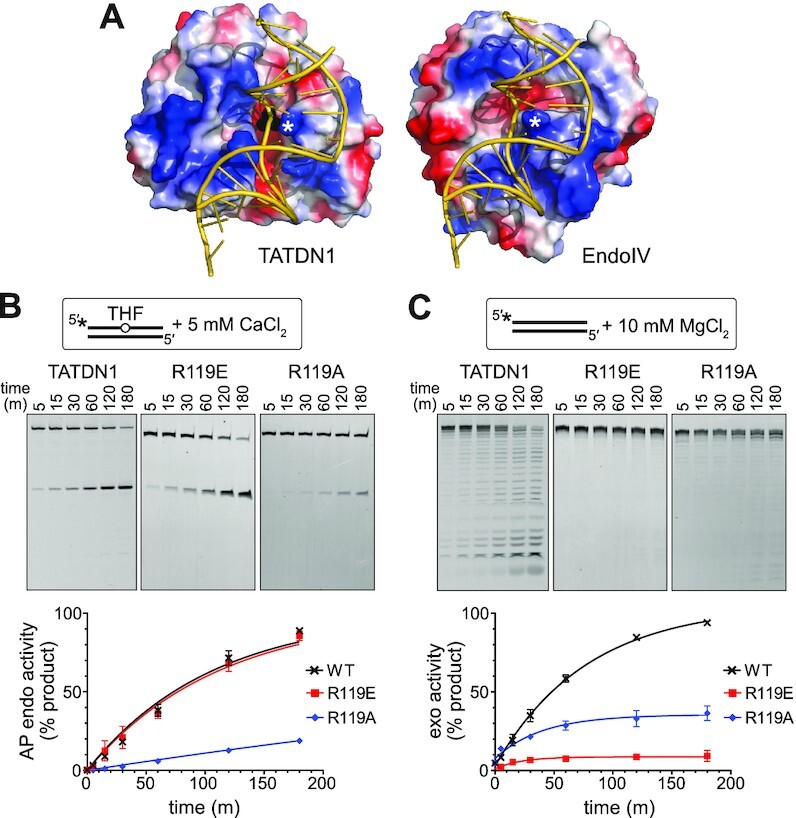
Biochemical characterization of the Arg119 mutants of TATDN1. (**A**) Model of TATDN1 bound to DNA containing an AP site (left) compared to the crystal structure of EndoIV bound to AP-DNA (right, PDB ID 2NQJ). The phosphate group of the dAMP bound in the active site of TATDN1 is shown in black spheres. The white asterisks denote the positions of Arg119 of TATDN1 and Arg37 of EndoIV. Protein surfaces are colored by electrostatic potential (red, positive; blue, negative). (**B**, **C**) Effect of Arg119 mutants on AP endonuclease (**B**) and exonuclease (**C**) activities, as monitored by denaturing PAGE. Data are quantified below the gels (mean ± SD, *n* = 3). (**B**) Incision of a 25-mer DNA substrate containing a THF in position 12 in the presence of 5 mM CaCl_2_. (**C**) Cleavage of an unmodified 25-mer DNA substrate in the presence of 10 mM MgCl_2_.

To gain insight into the manner in which TATDN1 binds DNA, we generated a model of DNA bound to TATDN1 by taking advantage of the similarity in protein fold between TatD and EndoIV (Supplementary Figure S8). Attempts to dock ideal B-form DNA to within reasonable proximity (<5 Å) of the metal cofactors without significant steric clashes between protein and DNA were unsuccessful. We were, however, able to dock the kinked AP-DNA from the co-crystal structure of EndoIV (62) as a rigid body with no steric clashes. The model predicted that Arg119 is important to stabilize the bound DNA by intercalating into the duplex in a manner similar to Arg37 in EndoIV (Figure 5A). Consistent with the importance of intercalation of a bulky side chain into the DNA, substitution of Arg119 to alanine, but not glutamate, severely reduced the AP endo activity of TATDN1 in double-stranded DNA (Figure 5B). In contrast, both alanine and glutamate mutants reduced the 3′-exo activity, which is operative on ssDNA (Figure 5C). The effect of R119E on 3′-exo activity suggests that this activity requires an interaction between the side chain and the DNA backbone.

### AP endonuclease activity is evolutionarily conserved

**Figure 6.**
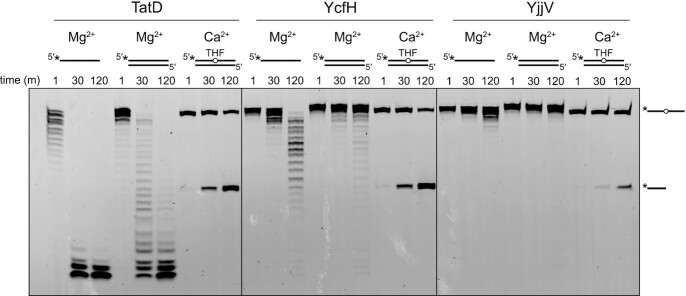
TatD AP endonuclease activity is conserved across evolution. Denaturing gel showing the activity of the *E. coli* TatD paralogs on three different DNA substrates in the presence of 10 mM MgCl_2_ or 5 mM CaCl_2_.

To determine whether the AP endonuclease activity is conserved among other TatD enzymes across evolution, we tested the exonuclease activities of the three *E. coli* TatD orthologs—TatD, YcfH and YjjV—using the same ssDNA and dsDNA substrates as we did for TATDN1 and TATDN3 (Figure 6A). Both TatD and YcfH showed the same 3′-exo specificity for ssDNA over dsDNA and robust AP endo activity on dsDNA as observed from the human orthologs. YjjV had very little nuclease activity, which we attribute to the low solubility of the protein, although we were able to see 3′-exo activity on ssDNA and AP endo activity on dsDNA. In general, the difference in exonuclease activities between ssDNA and dsDNA was even more pronounced for the *E. coli* proteins as compared with the human orthologs.

A previous study showed that deletion of TatD increased *E. coli* sensitivity to treatment with the oxidizing agent H_2_O_2_, but not with the alkylating agent methyl methanesulfonate (MMS), the crosslinking agents mitomycin C, or UV radiation (35). Given our finding that YcfH and YjjV also have AP endo and 3′-exo activities, and thus could have compensated for the loss of TatD in the previous study, we tested the sensitivity of a mutant E. coli strain lacking all three TatD paralogs (Δ*tatD*Δ*yjjV*Δ*ycfH*) toward various DNA damaging agents. EcTatD, YjjV and YcfH can be deleted without affecting cell growth under normal conditions (33). The results from the triple knockout were essentially the same as the single ΔtatD strain (Supplementary Figure S9). The Δ*tatD*Δ*yjjV*Δ*ycfH* mutant increased cellular sensitivity to the oxidizing agent H_2_O_2_ but not to UV radiation, and showed only a very modest increased sensitivity to MMS. These results are consistent with the previous study that the bacterial TatD enzymes are involved in oxidative DNA repair (35).

## DISCUSSION

This work shows that both human and bacterial TatDs contain AP endo activity in addition to the previously described 3′-exo activities of this nuclease family. Eukaryotic APE1 and APE2 and bacterial ExoIII and EndoIV AP endonucleases also exhibit 3′-exo activities, albeit with a different specificity than TatD. TatD’s exo activity is more active on ssDNA and is highly processive, whereas the other known AP endonucleases are more active on dsDNA and—with the exception of ExoIII—contain relatively non-processive 3′-diesterase and 3′-phosphatase activities that remove a single blocked or mismatched nucleotide (23,24,78,79). Recent work has provided mechanistic insight into how the endo and exo nuclease activities of APE1, APE2 and ExoIII are regulated within a single active site (80–83). Substrate specificity is largely dictated by a hydrophobic binding pocket that cradles the flipped AP site adjacent to the metal-bound active site (80,84,85), and higher exo and lower AP endo activities seem to be correlated with fewer aromatic residues within this pocket that would sterically exclude conformationally and chemically diverse 3′-terminal substrates (80,86). Based on this hypothesis, the robust exo activities of TATDN1 and TATDN3 may be explained by the fact that their putative AP site binding pockets do not contain any aromatic residues. Moreover, the exo activities that access 3′-ends in dsDNA in the APE/ExoIII enzymes largely rely on fraying of the duplex by intercalating residues to expose the 3′-end (81–83,87). In contrast, TATDNs likely require distinct ssDNA and dsDNA binding modes to enable the processive exonuclease activity on ssDNA and AP site specificity within dsDNA. Indeed, mutation of Arg119, predicted to penetrate dsDNA to stabilize an extrahelical (flipped) AP site, affects AP endo and 3′-exo activities differently.

TATDN1 (Class I) proteins differ from TATDN2/N3 (Class II) by the absence of two active site histidines, both of which play a role in binding a catalytic metal ion. While it seems clear from structural data that Class II proteins bind two metal cofactors (Supplementary Table S3), the number of catalytic metals employed by Class I proteins has until now been unclear. Our crystal structure and mixed-metal titration data both suggest that at least two metals are needed for catalysis by human TATDN1. In a two-metal mechanism, one metal (Me_A_^2+^) activates a water molecule to perform a nucleophilic attack on the scissile phosphate, creating a pentavalent transition state that is stabilized by the second metal ion (Me_B_^2+^). Catalysis by a single metal cofactor would require an amino acid residue to act as a general base to activate the water nucleophile (74), and we have no evidence of such a residue as all the active site mutants we tested retained some residual nuclease activity. We also do not have evidence of a third catalytic metal (35). A putative third metal binding site defined by His174, Ser175, Asn196, and Glu220 is unoccupied in our structure, and mutation of Glu220 or His174 in TATDN1 did not have a significant effect of either nuclease activity. While our data are consistent with a two-metal mechanism, we cannot rule out that TATDN1 uses a one-metal mechanism (84), in part because putative catalytic water nucleophile was not present in our structure. A water nucleophile would most likely reside in the more solvent exposed of the two Me_A_^2+^ positions that we observed in our structure. We speculate that in TATDN1 Me_A_^2+^ is bound only when DNA is present since it partially occupies two positions and mediates interactions between the DNA phosphate and the protein in our structure. In contrast, Me_B_^2+^ is fully occupied and coordinated entirely by protein side chains and therefore its binding likely is not as transient.

The number of known AP endonucleases in humans and in *E. coli* is now five each (assuming that human TATDN2 also contains such activity). The most important outstanding questions now are—what is the role of the AP endo activity in cells and how do the AP endo and 3′-exo activities contribute to the various functions observed in multiple organisms? Both are consistent with TatDs acting in DNA repair capacity, as implicated by the ability of the *E. coli* enzymes to protect against oxidative stress (35). In support of a DNA repair role, all three human TATDNs are all localized to the nucleus (88). Moreover, the dual AP endo/3′-exo activities and the relatively low AP endo activity of recombinant TATDN proteins compared to APE1 and EndoIV (89) are reminiscent of APE2, and suggest that TATDN proteins do not play a role in BER but have more specialized repair functions. Indeed, the three *E. coli* TatD paralogs, while clearly important for oxidative stress, are not essential since cells lacking TatD, YjjV, and YcfH are viable under aerobic or anaerobic growth conditions (33). Nevertheless, TatDs are highly conserved across evolution and are often present in multiple copies. While the exonuclease activity could serve to repair damaged 3′-ends, it could also disrupt other processes or lead to loss of genetic information if unregulated (35,90). Thus, it is likely that other factors regulate TATDN’s two relatively equally efficient nuclease activities in cells. Both AP endo and 3′-exo activities could contribute to the cell cycle and developmental phenotypes in zebrafish TATDN1 knockouts (39), as well as the DNA fragmentation during apoptosis in yeast, *C. elegans* and *Trypanosoma brucei* (36–38). Additionally, the endonuclease activity previously reported for zebrafish TATDN1 that enables decatenation of plasmid DNA (39) may in fact be the result incision at AP sites present in those substrates.

Several studies have reported that TATDN1 overexpression in cancers is associated with poor prognosis (43–47). TATDN1 overexpression promotes cell proliferation and migration in non-small cell lung cancer (NSCLC), and induces resistance to cisplatin treatment (43,45). These studies focused on the effects of RNA transcript, and thus it is not clear if the proteins themselves are involved. Wang *et al.* (45) provided immunohistochemical evidence for protein expression during NSCLC, but further studies are necessary to assess whether the enzymatic activities of the human TatD proteins are involved in cisplatin resistance in NLCLC. Despite these outstanding questions, our discovery of a second nuclease activity in the TatD family of enzymes and molecular characterization of AP endo and 3′-exo activities provide a foundation for further studies to elucidate their biological roles.

## DATA AVAILABILITY

Structure factors and coordinates have been deposited in the Protein DataBank under accession number 8EFG. All other data is available from the authors upon request.

## Supplementary Material

gkad133_Supplemental_File
