# Supplementary material for: Human and bacterial TatD enzymes exhibit apurinic/apyrimidinic (AP) endonuclease activity
Source: Nucleic Acids Res. 2023 Mar 7;51(6):2838–49. doi: 10.1093/nar/gkad133 (PMC10085689; doi:10.1093/nar/gkad133)
Supplement: gkad133_Supplemental_File [file gkad133_supplemental_file.pdf]

Supplemental Information

Human and bacterial TatD enzymes exhibit apurinic/apyrimidinic endonuclease activity

Jonathan Dorival[1] and Brandt F. Eichman[1,2,*]

[1]Department of Biological Sciences, Vanderbilt University, Nashville, Tennessee, USA

[2]Department of Biochemistry, Vanderbilt University School of Medicine, Nashville, Tennessee, USA

*Corresponding author: brandt.eichman@vanderbilt.edu

Supplemental Tables

Table S1. Sequences of oligonucleotides used

Name	Sequence (5'→3')
ss5	FAM-CGTCTCCTGAACTGTGCTATCGCTC
ss3	CGTCTCCTGAACTGTGCTATCGCTC-FAM
ss5THF	FAM-CGTCTCCTGAAC/(THF)/GTGCTATCGCTC
ss5dU	FAM-CGTCTCCTGAAC/(dU)/GTGCTATCGCTC
comp	GAGCGATAGCACAGTTCAGGAGACG
comp3ovh	GCACAGTTCAGGAGACG
comp5ovh	AAAAAAAAGAGCGATAGCACAGTTCAGGAGACG
	ss5
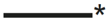	ss3
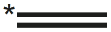	ss5 + comp
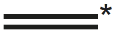	ss3 + comp
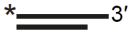	ss5 + comp3ovh
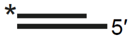	ss5 + comp5ovh
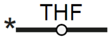	ss5THF
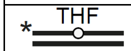	ss5THF + comp

Table S2. X-ray data collection and refinement statistics [a]

**Data collection**	
Space group	$P2_12_12_1$
Cell dimensions	
a, *b*, *c* (Å)	55.88 76.51 77.36
α, *β*, *γ* (°)	90.0 90.0 90.0
Resolution (Å)	45.30 - 1.50 (1.55 - 1.50)
R_{meas}	0.06 (0.89)
Avg. $I/\sigma I$	15.93 (2.53)
$CC_{1/2}$	0.99 (0.78)
Completeness (%)	98.4 (86.4)
Redundancy	6.6 (6.6)
Wilson *B*-factor ($Å^2$)	21.4
**Refinement**	
Resolution (Å)	45.30 - 1.50 (1.54-1.50)
No. reflections	52957 (3536)
R_{work}	0.1627 (0.2820)
R_{free} [b]	0.1888 (0.2870)
No. atoms [c]	2887
Protein	2424
ligands	46
Water	417
Avg. *B*-factors [c] ($Å^2$)	25.54
Protein	23.10
ligands	29.19
Water	39.32
R.m.s. deviations	
Bond lengths (Å)	0.010
Bond angles (°)	1.54

[a] Statistics for the highest resolution shell are shown in parentheses.
[b] R_{free} was determined from the 5% of reflections excluded from refinement.
[c] Riding hydrogen atoms were not included in no. atoms or avg. *B*-factors.

Table S3. Metal ions and ligands present in TatD crystal structures

Group[a]	Organism	Name	Metal	Ligand	PDB ID
I	*Homo sapiens*	TATDN1	2 Zn^{2+}	dAMP	8EFG (this work)
I	*Homo sapiens*	TATDN1	-	-	2XIO
I	*Saccharomyces cerevisiae*	TatD	1 Mg^{2+}	-	3E2V
I	*E. coli*	TatD	1 Zn^{2+}	-	1XWY
I	*E. coli*	TatD	-	-	4P5U
I	*E. coli*	TatD	-	d(GCT)	4PE8
I	*Entamoeba histolytica*		-	-	3IPW
I	*Pseudomonas putida*		1 Zn^{2+}	Citrate	3RCM
II	*Homo sapiens*	TATDN3	2 Zn^{2+}	PO_4^{2-}	2Y1H
II	*E. coli*	YjjV	2 Zn^{2+}	PEG	1ZZM
II	*E. coli*	YcjH	2 Zn^{2+}	-	1YIX
II	*Staphylococcus aureus*	MW0446[b]	2 Ni^{2+}	-	2GZX
II	*Staphylococcus aureus*	MW0446[b]	2 Ni^{2+}	PO_4^{2-}	6L25
II	*Thermatoga maritima*	TM0667	-	-	1J6O
II	*Archeoglobus fulgidus*	AF1765	2 Zn^{2+}	-	3GUW
II	*Deinococcus radiodurans*		1 Mn^{2+}	MPD	3GG7

[a] Group I and II refer to the presence of (G/A)xN and HxH active site motifs, respectively
[b] MW0446 has also been designated SAV0491 and SaTatD

Table S4. AP endo- and exonuclease rates of TatDN1 mutants [a]

TatDN1	AP endo k (10^{-2} min^{-1})	fold change (relative to WT)	Exo k (10^{-2} min^{-1})	fold change (relative to WT)
WT	1.00 ± 0.08	1	1.34 ± 0.14	1
H77A	0.12 ± 0.01	0.1	1.80 ± 0.08	1.3
E112A	0.33 ± 0.02	0.3	0.40 ± 0.01	0.3
E112Q	2.02 ± 0.44	2.0	5.07 ± 0.28	3.8
R119A	0.12 ± 0.01	0.1	0.25 ± 0.05	0.2
R119E	0.98 ± 0.10	1	n.d. [b]	
H149A	0.44 ± 0.01	0.4	1.15 ± 0.08	0.9
R151A	0.53 ± 0.02	0.5	1.54 ± 0.07	1.1
H174A	0.40 ± 0.03	0.4	1.15 ± 0.32	0.9
E220A	1.33 ± 0.06	1.3	1.83 ± 0.07	1.4
D222A	0.62 ± 0.13	0.6	1.59 ± 0.55	1.2

[a] Rates are mean ± S.D. for three independent measurements
[b] n.d., none detected

Table S5. AP endo- and exonuclease rates of TatDN3 mutants [a]

TatDN3	AP endo k (10^{-2} min^{-1})	fold change (relative to WT)	Exo k (10^{-2} min^{-1})	fold change (relative to WT)
WT	5.08 ± 0.25	1	5.69 ± 0.30	1
E107A	2.28 ± 0.01	0.4	1.20 ± 0.42	0.2
E107Q	15.74 ± 0.71	3.1	28.05 ± 5.17	4.9
D218A	0.16 ± 0.03	0.03	0.20 ± 0.15	0.04

[a] Rates are mean ± S.D. for three independent measurements

Supplemental Methods

Protein production from BH110 cells. For expression in BH110 (*nfo::kan*^{R} [Δ(*xth-pncA*)*90 X::Tn10*]) cells (1), TATDN1 and TATDN3 were subcloned into a pOKD5 expression vector (Amp^R) encoding a N-terminal Rhinovirus 3C-cleavable hexahistidine-SUMO fusion tag. Plasmids were transformed into *E. coli* BH110(DE3) cells and proteins expressed and purified the same as those in BL21(DE3) as described in the main text.

Cell growth assays. Wild-type and Δ*tatD*Δ*yjjV*Δ*ycfH* variant MC4100 *E. coli* strains (2) used to perform the cell growth studies were kindly provided by Professor Tracy Palmer and Dr. Felicity Alcock (Newcastle University). Precultures were grown overnight in LB medium at 37°C. Precultures of the wild-type and triple mutant MC4100 strain were diluted to reach the same cell density measured at 600 nm (OD_{600}). Cultures were then diluted 100-fold in 200 µL of LB medium containing various concentrations of methyl methanesulfonate (MMS) or hydrogen peroxide (H_2O_2). Cultures were incubated in 96-well, flat-bottom plates with shaking for 18 h using a Synergy 2 multi-detector microplate reader (BioTek). Cell density was measured at 600 nm every hour. Experiments were performed in triplicate. For growth experiments on solid medium (spot assays), precultures were grown as described above, diluted 10- to 10^5-fold in LB medium, and 5 µL of the dilution immediately spotted on LB agar plates. Plates were exposed to UV-C radiation (up to 6 mJ/cm^2) using a Stratalinker 1800 UV Crosslinker (Stratagene) and incubated at 37°C for 18 h before being imaged.

1. Ishchenko, A.A., Sanz, G., Privezentzev, C.V., Maksimenko, A.V. and Saparbaev, M. (2003) Characterisation of new substrate specificities of Escherichia coli and Saccharomyces cerevisiae AP endonucleases. *Nucleic Acids Res*, **31**, 6344-6353.
2. Wexler, M., Sargent, F., Jack, R.L., Stanley, N.R., Bogsch, E.G., Robinson, C., Berks, B.C. and Palmer, T. (2000) TatD is a cytoplasmic protein with DNase activity. No requirement for TatD family proteins in sec-independent protein export. *J Biol Chem*, **275**, 16717-16722.

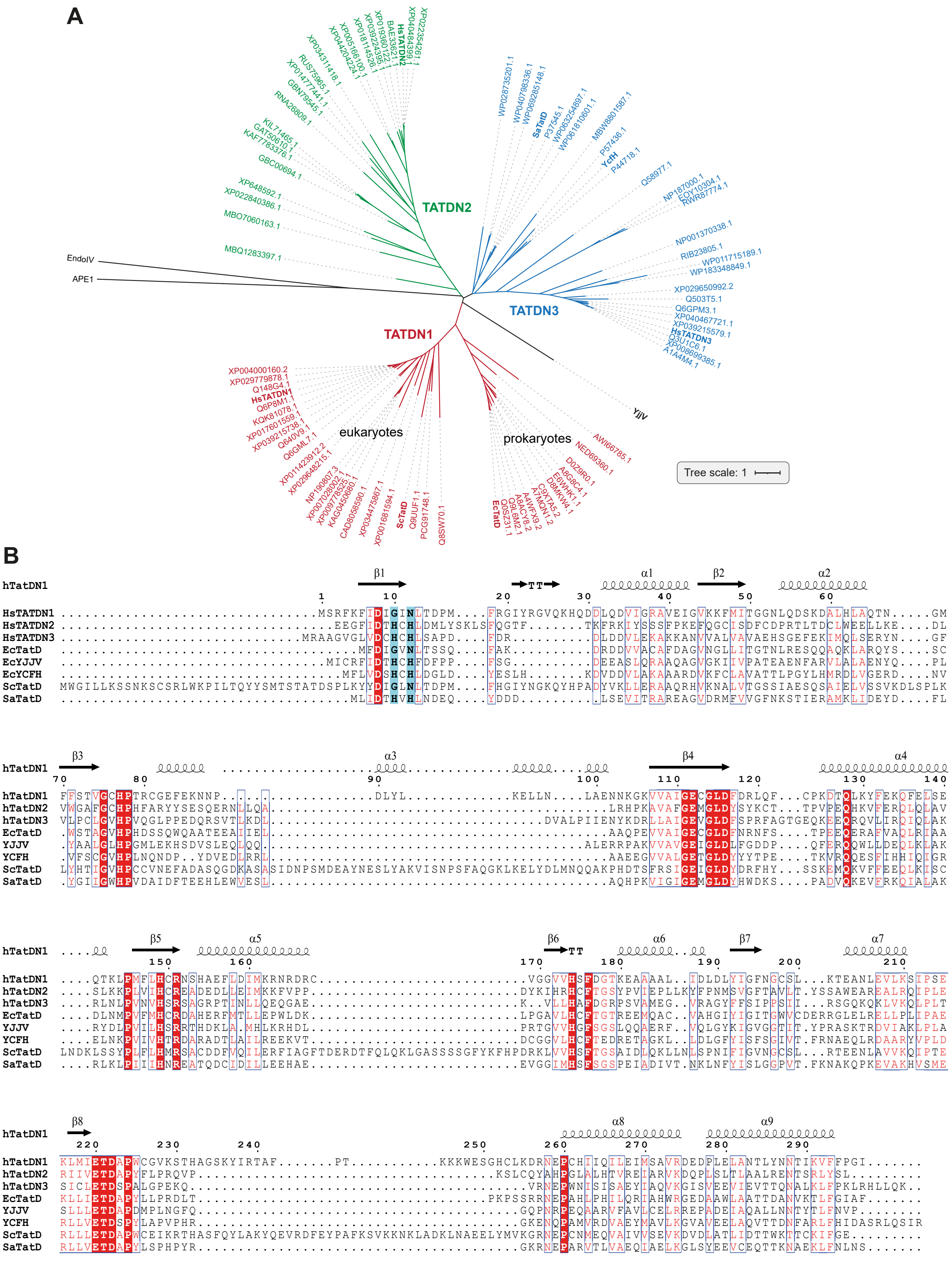

Figure S1. Sequence alignment of TatD proteins. (A) Phylogenetic tree of TatD proteins. (B) Sequences from *Homo sapiens* (Hs), *Escherichia coli* (Ec), *Saccharomyces cerevisiae* (Sc), and *Staphylococcus aureus* (Sa) were aligned using Clustal Omega and plotted using ESPript (https://espript.ibcp.fr). The A/GxN and HxH motifs are highlighted blue. Secondary structure of human TATDN1 is displayed above the alignment.

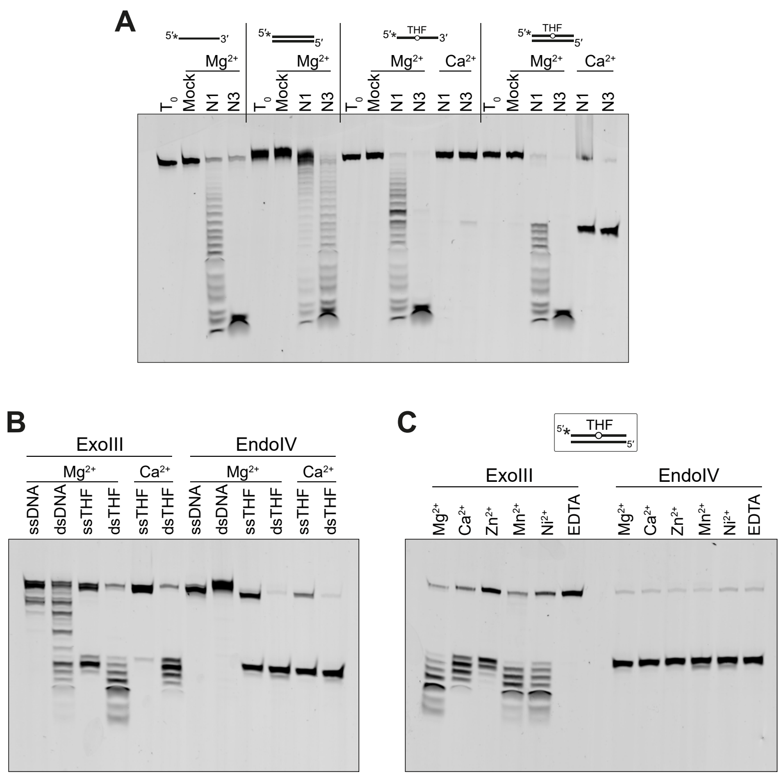

Figure S2. TATDN AP endo activity does not result from ExoIII or EndoIV contamination. (A) Nuclease activities of TATDN1 and TATDN3, purified from the *xth⁻ nfo⁻ E. coli* strain BH110(DE3). Reactions contained 10 mM $MgCl_2$, 100 nM DNA, and 20 μM enzyme, were performed at 37°C for 3 h, and visualized by denaturing gel electrophoresis. T_0, substrate only, no reaction; Mock, no enzyme control reaction for 3 h. (B) Nuclease activities of ExoIII or EndoIV on the same DNA substrates shown in panel A. Reactions containing 50 nM ExoIII or 8 nM EndoIV, 100 nM DNA and either 10 mM Mg^{2+} or 5 mM Ca^{2+} were incubated at 37°C for 30 m. (C) Metal dependence of ExoIII and EndoIV. Reactions contained 50 nM ExoIII or 8 nM EndoIV and either 10 mM $MgCl_2$, 5 mM $CaCl_2$, 0.5 mM $ZnCl_2$, 3 mM $MnCl_2$, 1 mM $NiCl_2$, or 10 mM EDTA. Reactions were performed at 37°C for 30 m. For B and C, ExoIII and EndoIV were purchased from New England Biolabs and were incubated for 1 h in the presence of 5 mM EDTA prior to start the reaction to ensure no contaminant metals were introduced.

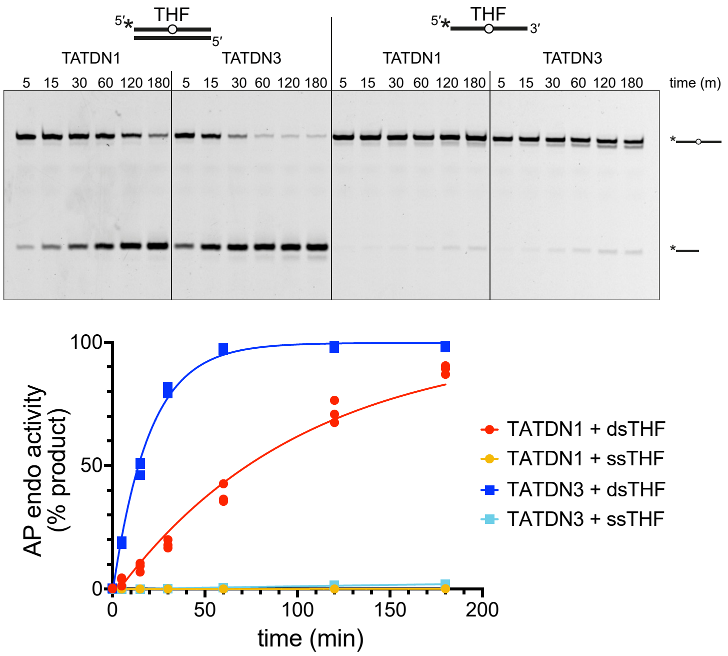

Figure S3. AP endonuclease kinetics. The AP endonuclease activities of TATDN1 and TATDN3 on double-stranded or single-stranded DNA containing a central tetrahydrofuran AP analog (THF) was monitored by denaturing gel electrophoresis under single-turnover conditions. Reactions were carried out at 37°C and contained 20 mM EPPS pH 8.0, 50 mM NaCl, 5 mM $CaCl_2$, and 2.5% glycerol. Intensities of bands on the gel are quantified in the plot at the bottom. Rate constants (k_{cat}) for THF incision in dsDNA were determined from exponential fits to the data (TATDN1=(1.0 ± 0.1) ×10^{-2} min^{-1}; TATDN3=(5.1 ± 0.3) ×10^{-2} min^{-1}).

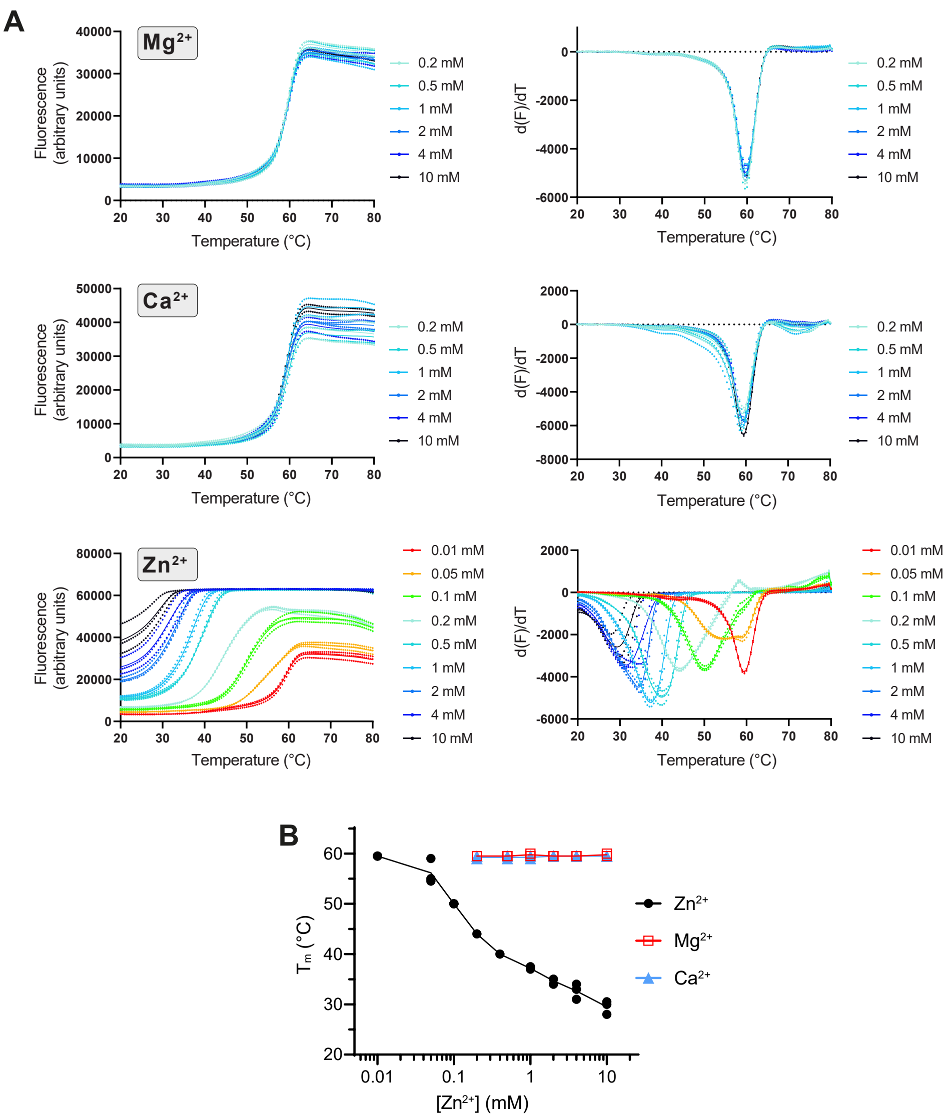

Figure S4. Metal-dependence on TATDN1 stability. (A) Differential scanning fluorescence thermal denaturation profiles for TATDN1 in the presence of either Mg^{2+}, Ca^{2+}, or Zn^{2+} are shown on the left. Samples contained 15 μM TATDN1, 30 mM HEPES pH 7.5, 100 mM NaCl, and 0.5X SYPRO Orange in addition to the divalent cations. Plots on the right are first derivatives of the thermal denaturation data used to calculate melting temperatures (T_m). (B) T_m as a function of ion concentration.

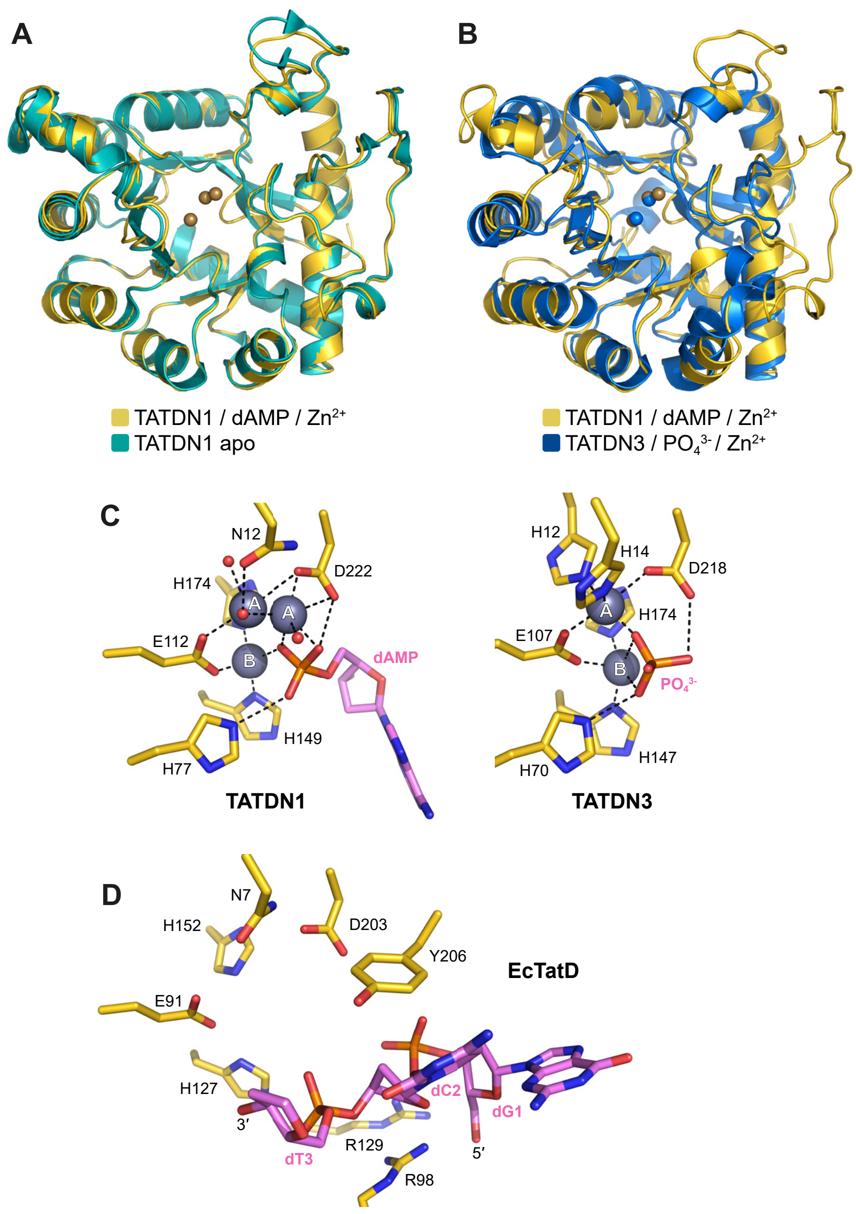

Figure S5. Structural similarity between human TATDN proteins. (A) Structural alignment of apo TATDN1 (PDB ID 2XIO, cyan) and in complex with Zn^{2+}/dAMP (gold). Zinc ions are shown as spheres. (B) Structural alignment of TATDN1/Zn^{2+}/dAMP (gold) and TATDN3 Zn^{2+}/PO_4^{3-} (PDB ID 2Y1H, blue) complexes. (C) Comparison human TATDN1 and TATDN3 active sites (same proteins as in panel B). Protein and DNA carbons are gold and pink, respectively. Zn^{2+} ions and water oxygens are shown as grey and red spheres, respectively. (D) EcTatD bound to d(GCT) trinucleotide (PDB ID 4PE8) shown in the same orientation as TATDN1 in panel C.

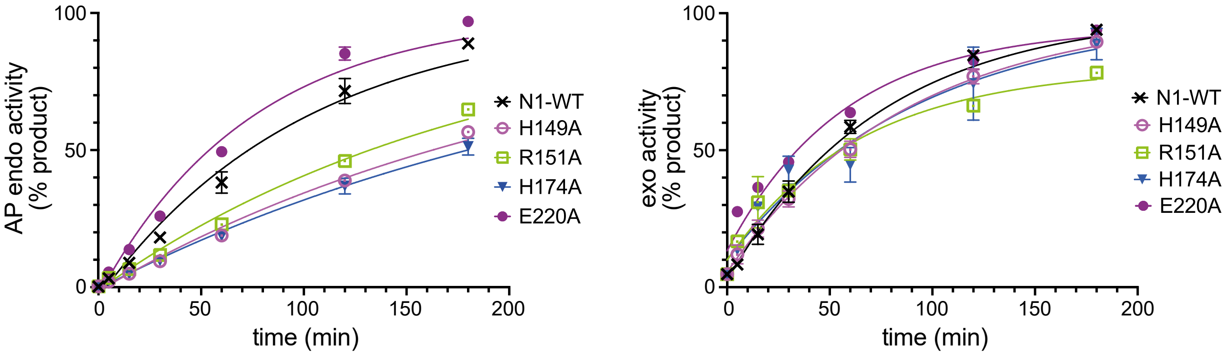

Figure S6. Mutational analysis of TATDN1. AP endonuclease (left) and exonuclease (right) activity of TATDN1 mutants. AP endonuclease activity was measured on a 25-mer THF-containing DNA duplex in the presence of 5 mM $CaCl_2$ and the exonuclease activity was measured using an unmodified 25-mer DNA duplex in the presence of 10 mM $MgCl_2$. Data are represented as the mean ± SD (n=3). Representative gels are shown in Fig. S7.

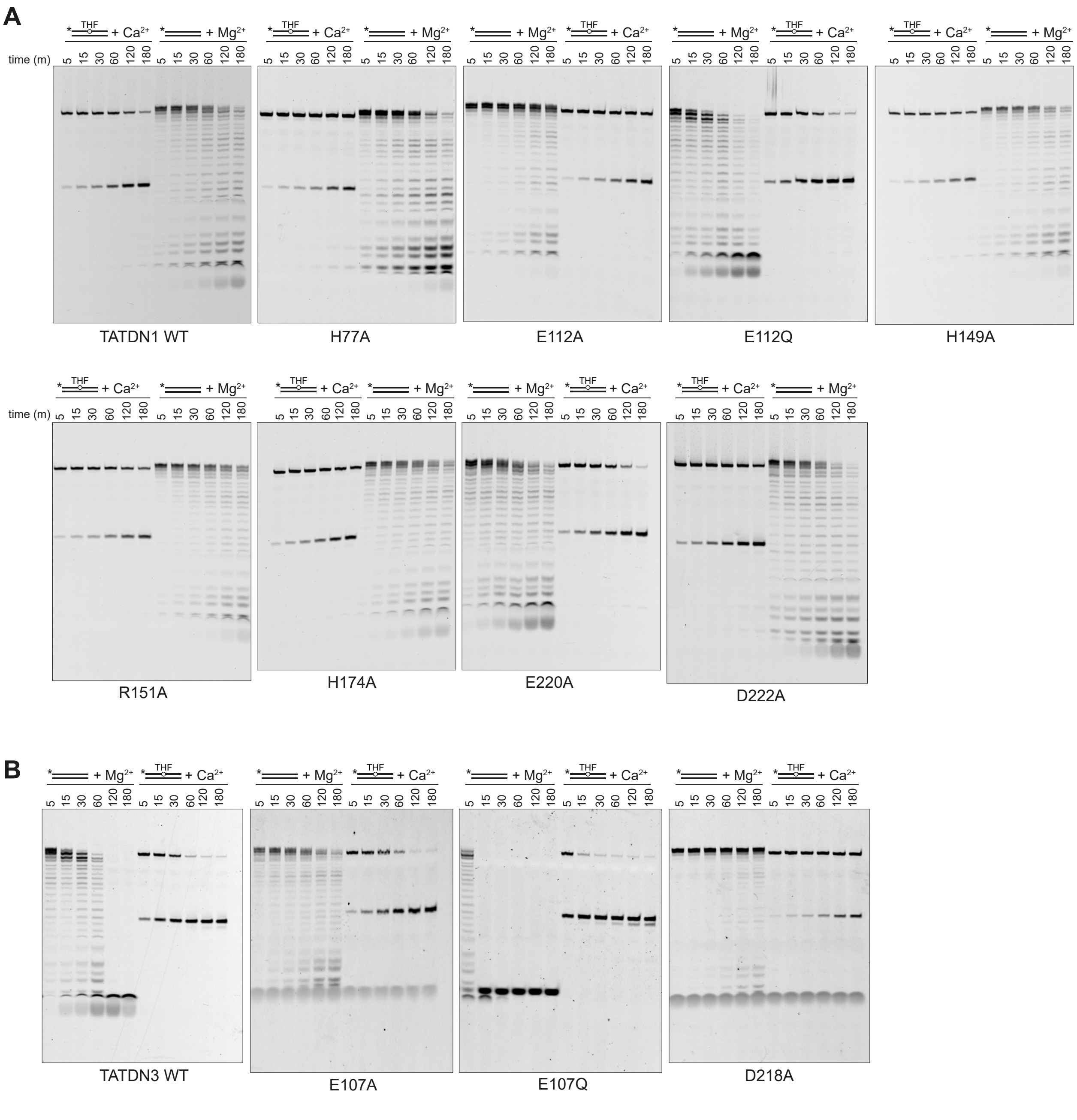

Figure S7. AP endo and 3′-exo activities of TATDN1 and TATDN3 mutants. Representative raw data for TATDN1 (A) and TATDN3 (B) active site mutants. Data from three such experiments are quantified in Fig. 4. AP endonuclease activity was measured on a 25-mer THF-containing DNA duplex in the presence of 5 mM $CaCl_2$ and the exonuclease activity was measured using an unmodified 25-mer DNA duplex in the presence of 10 mM $MgCl_2$. Asterisks denote the position of a FAM label at the 5′ end.

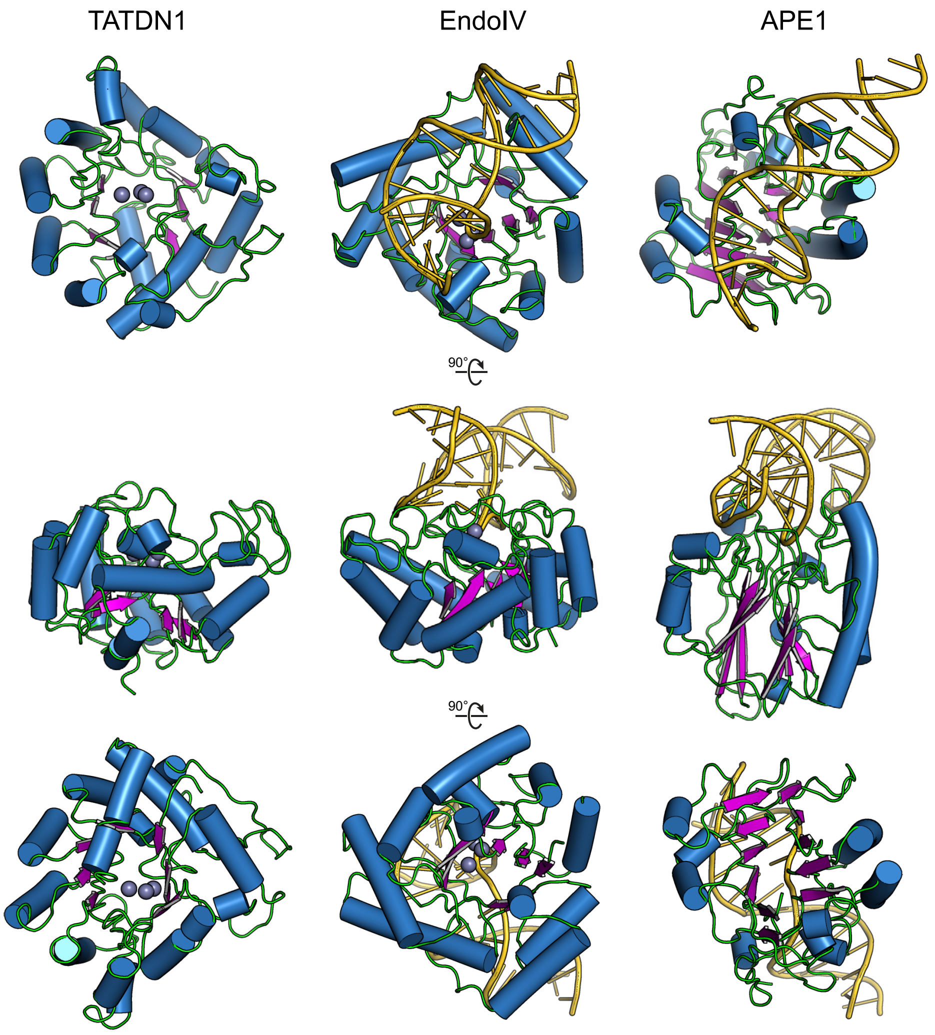

Figure S8. Crystal structures of AP endonucleases. Orthogonal views of human TATDN1 (left), *E. coli* EndoIV bound to AP-DNA (PDB ID 2NQJ), and human APE1 bound to AP-DNA (PDB ID 1DEW). DNA is shown in gold and proteins are colored according to secondary structure (blue helices, magenta sheets, green coils).

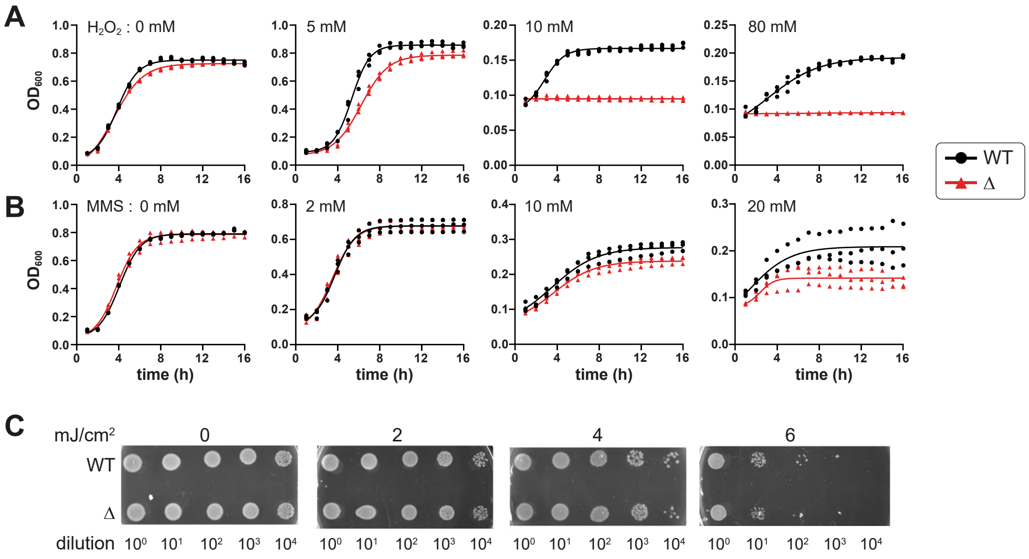

Figure S9. TatD paralogs sensitize *E. coli* to oxidation stress. (A,B) Growth curves of wild-type (WT) and Δ*tatD*Δ*yjjV*Δ*ycfH* (Δ) *E. coli* in the presence of H_2O_2 (A) and MMS (B) at the specified concentrations. (C) Sensitivity to UV-C radiation at the indicated doses.
